# Are Energy and Protein Intakes Lower Than Requirements in Older Adults? An Urgent Issue in Hospitals and Nursing Homes

**DOI:** 10.3390/nu15153307

**Published:** 2023-07-26

**Authors:** Marie Blanquet, Candy Guiguet-Auclair, Pauline Berland, Guillaume Ducher, Anaïs Sauvage, Sylvain Dadet, Vincent Guiyedi, Nicolas Farigon, Jérôme Bohatier, Laurent Gerbaud, Yves Boirie

**Affiliations:** 1 Unité de Recherche Clinique, CH Mauriac, 15200 Mauriac, France; v.guiyedi@ch-mauriac.fr; 2 CNRS, Institut Pascal, Université Clermont Auvergne, CHU Clermont-Ferrand, 63000 Clermont-Ferrand, France; cauclair@chu-clermontferrand.fr (C.G.-A.); p_berland@chu-clermontferrand.fr (P.B.); lgerbaud@chu-clermontferrand.fr (L.G.); 3 Service de Santé Publique, CHU Clermont-Ferrand, 63000 Clermont-Ferrand, France; 4 Gériatrie, Clinique La Châtaigneraie, 63110 Beaumont, France; guillaume.ducher@elsan.care; 5 Recherche et Développement, Nutriset, 76770 Malaunay, France; asauvage@nutriset.fr; 6 Médecine, CH Brioude, 43100 Brioude, France; sdadet@ch-brioude.fr; 7 Nutrition Clinique, CHU Clermont-Ferrand, 63000 Clermont-Ferrand, France; nfarigon@chu-clermontferrand.fr (N.F.); yboirie@chu-clermontferrand.fr (Y.B.); 8 Gériatrie, CH Riom, 63000 Clermont-Ferrand, France; jbohatier@chu-clermontferrand.fr; 9 Unité de Nutrition Humaine, Université Clermont Auvergne, INRA, UMR 1019, 63000 Clermont-Ferrand, France

**Keywords:** energy intake, hospitalization, malnutrition, nursing home, nutrient gap, nutritional assessment, older adults, protein intake

## Abstract

Energy and protein intakes lower than requirements are associated with worsening health outcomes. Here we set out to evaluate gaps between energy and protein intakes and requirements in older adults in hospitals and in nursing homes (NH). A cross-sectional study included 360 inpatients and residents aged 75 years and older in two acute care wards; i.e., a multidisciplinary care unit (MCU) and a geriatric care unit (GCU), a geriatric rehabilitation unit (GRU), and two NH. Intakes were measured for three days. Requirements were based on French National Health Authority recommendations. Energy and protein intakes were under the minimum requirement of 30 kcal/kg/day and 1.2 g/kg/day in 89.5% and 100% of MCU patients, respectively, 75.5% and 64.2% of GCU patients, 92.7% and 90.9% of GRU patients, and 83.8% and 83.8 of NH residents. Intake-to-requirement gaps were not significantly associated with malnutrition, except in the GCU group where non-malnourished patients had higher energy gaps than malnourished patients. Intakes fell dramatically short of requirements in older adults in both hospital and NH settings irrespective of malnutrition status. A new paradigm based on a patient-centered approach should be developed to adapt meals served in hospital and in NH.

## 1. Introduction

Nutritional intake in older adults is a major issue that healthcare professionals should be systematically addressing whatever the patient’s setting, whether at home, in hospital, or in nursing homes (NH) care [1,2,3]. Energy and protein intakes lower than requirements are associated with worsening health outcomes such as frailty, impaired muscle function, healthcare-associated infections, mortality, longer hospital stays, and frequent readmissions [4,5,6,7,8,9,10,11,12].

Studies have measured food intakes in patients hospitalized in acute care wards, but rarely in rehabilitation units or NH and specifically in older adults [11,13,14,15,16,17,18,19,20,21]. Most of these studies estimated food intake based on percentage intake of meal served (0%, 25%, 50%, 75%, or 100%) or on the larger threshold of meal served (<100%, <75% or ≤50%). Three studies calculated energy intake (kcal) and protein intake (g) based on food consumed and compared the results against inpatient requirements [13,14,17]. In these three studies, two were located in the same hospital, using the same structure survey protocol at two different times (1999 and 2008) [13,14]. They aimed to measure the effect of a nutrition permanent improvement process launch in the hospital by measuring 24 hours food intakes and requirements [13, 21]. Measurements were performed by dieticians [14]. Sanson et al. used photography to assess intakes and estimated requirements in older acute-care patients (81.5 ± 11.5 years old) over the first 5 days of a hospital stay [17]. Two further studies estimated intakes based on patient interviews and calculated the proportion of patients that failed to meet their energy and/or protein requirements: Beavan et al. [15] studied patients with a median age of 72 years (range 22–98) in 25 medical and surgical wards, and Vasse et al. [19] measured protein intakes in acute medicine or surgical wards in patients aged at least 65 years old (mean age was 77.4 ± 5.6 in the low-malnutrition-risk group and 78.7 ± 6.4 in the medium/high-risk group). 

However, to our knowledge, no study has calculated the gaps between energy/protein intakes and requirements in a geriatric population hospitalized in acute-care and rehabilitation wards and in NH. Previous studies have analyzed factors associated with low intakes, but not the extent of the gaps.

Here, to address this issue, we evaluated energy and protein gaps between intakes and requirements in older adults hospitalized in acute and rehabilitation wards or living in NH with long-term care facilities, in order to compare intake-to-requirement gaps according to malnutrition status, and to identify others factors associated with these gaps.

## 2. Materials and Methods

### 2.1. Study Design

A multidepartment cross-sectional study was conducted in the Auvergne region (south-central France) between September 2017 and December 2018 [22]. The units concerned were community hospital units—a multidisciplinary care unit (MCU) and a geriatric rehabilitation unit (GRU)—and a university hospital geriatric care unit (GCU). We also included each NH that had long-term care facilities at these two hospitals. The study was proposed to MCU, GCU, and GRU inpatients during the first 3 days of their hospital stay and to all NH residents. Readmitted patients could not be included a second time around. The Confusion Assessment Method was used to identify confused participants (inpatients or residents), and if any participant showed an acutely confused state or a medical history of dementia, then the study was presented to their primary care person. The primary care person was declared by the patient at the admission in hospital care wards or to the NH. If the patient was unable to declare a primary care person, healthcare professionals checked whether the care person declared at the most recent previous hospitalization remained unchanged. Inpatient data were collected during the first 7 days of the hospital stay, and NH resident data were collected during the 14 days following weight measurement. The study was registered at Clinical Trials.gov under number NCT03196622.

### 2.2. Participants

Inpatients and NH residents aged 75 years old and above were eligible for inclusion. Exclusion criteria were older adults in end-of-life care (death expected within the next 72 h), inappropriate medical situation (no expected health improvement through monitoring nutritional intakes, as the patients ate for pleasure), inpatient or resident refusal to participate, or refusal of the primary care person to participate if the inpatient or resident was found to show confusion or dementia, vulnerable older adults under guardianship, and older adults fed exclusively via percutaneous endoscopic gastrostomy.

### 2.3. Nutritional Intakes

Participant nutritional intakes were measured for 3 days as recommended by French guidelines [23]. The care assistant recorded the quantity eaten as 0%, 25%, 50%, 75%, or 100% per dish and per meal for breakfast, lunch, and dinner, plus any between-meal oral nutrition support (ONS) snacks. Three dieticians then calculated energy intakes in kilocalories (kcal) and protein intakes in grams (g) per day for each inpatient and resident that had a complete set of nutritional data. Inpatient energy and protein requirements were calculated based on the French National Health Authority guidelines; i.e., 30–40 kcal/kg/day for energy and 1.2–1.5 g/kg/day for protein [24]. Adjusted weight was calculated for body mass index (BMI) above 30 kg/m^2^ as adjusted weight = ideal weight (kg) + 0.25 [measured weight (kg) − ideal weight (kg)], where ideal weight (kg) = height (cm) − 100 − ([height (cm) − 150/] n), with n = 2.5 in women and n = 4 in men.

### 2.4. Malnutrition Diagnosis

The diagnosis of malnutrition was established according to the updated French National Health Authority guidelines that integrate the Global Leadership Initiative on Malnutrition (GLIM) criteria [25,26]. Participants were classified as malnourished if they met at least one phenotypic criterion and at least one etiological criterion. The phenotypic criteria were: unintentional weight loss (>5% in 1 month or ≥10% in 6 months), low BMI (<22 kg/m^2^), and low muscle mass corresponding to Fat-Free Mass Index (FFMI) < 15 kg/m^2^ in women and <17 kg/m^2^ in men. The etiological criteria were reduced food intake or assimilation (≤50% of energy requirements > 1 week, or any food intake reduction for >2 weeks, or any chronic gastrointestinal condition that adversely impacted food assimilation or absorption), or inflammation (related to acute disease/injury or chronic disease).

We considered that all older MCU, GCU, and GRU patients met the etiological criterion for inflammation, as they were all hospitalized for acute disease related or not to a chronic disease. We considered that older adults in NH who had a score of 3 or 4 on one of the Cumulative Illness Rating Scale for Geriatrics (CIRS-G) [27] organ-system categories (excluding the “eyes, ears, nose, throat, and larynx” category) met the etiological criterion for inflammation.

Severity of malnutrition was graded as either stage 1—moderate malnutrition, or stage 2—severe malnutrition, based on the following phenotypic criteria: weight loss (≥10% in 1 month or ≥15% in 6 months), low BMI (<20 kg/m^2^), albuminemia (≤30 g/L).

### 2.5. Measurements

Sociodemographic data were collected on age, gender, living situation (at home, in a NH, other), and life situation (alone, with partner, with a family member, other).

Anthropometric measurements for each participant included usual weight (kg), weight at admission (kg), height (m), and BMI (kg/m^2^). Usual weight was the patient’s regular weight when at home, and weight at admission was the weight measured by healthcare professionals on admission to care. Muscle strength was measured by handgrip strength (HGS) on a Jamar^®^ hydraulic hand dynamometer in sitting position following the Southampton protocol [28]. Muscle mass was estimated by FFMI based on bioelectrical-impedance analysis (BIA) using a Bodystat^®^ 1500 analyzer. Sarcopenia was defined according to the 2019 European Working Group on Sarcopenia in Older People (EWGSOP) revised criteria [29]. Probable sarcopenia was defined as low muscle strength, and sarcopenia was confirmed as low muscle strength combined with low muscle mass. The thresholds for low muscle strength were HGS < 16 kg for women and <27 kg for men. 

Oral daily medications were recorded by pharmaceutical molecule. 

Comorbidity was measured using the CIRS-G [27], which assesses comorbidity through 14 organ system categories on a severity scale, scored as follows: no problem (0), current mild problem or past significant problem (1), moderate disability or morbidity/requiring ‘first-line’ therapy (2), severe/constant significant disability/uncontrollable chronic problems (3), extremely severe/immediate treatment required/end-organ failure/severe functional impairments (4). The total CIRS-G score is the sum of each of the 14 individual organ system scores, ranging from 0 to 56. Severity index is the mean of the scores of the first 13 categories, excluding psychiatric impairment. Comorbidity index is the number of categories with a score of 2 or higher, including psychiatric impairment. 

The Katz Activities of Daily Living (ADL) scale [30] and the Lawton Instrumental Activities of Daily Living (IADL) scale [31] were used to measure inpatient functional ability. The IADL scale was not used in NH residents. The ADL scale explores six dimensions scored as 0 (unable), 0.5 (partially able), or 1 (able); i.e., for bathing, dressing, toileting, transferring, feeding, and continence, giving a total score from 0 to 6. The IADL scale measures 8 dimensions scored as 0 (unable) or 1 (able); i.e., for telephoning, shopping, cooking, housekeeping, laundry, transportation, managing medications, and managing finances, giving a total score from 0 to 8. For the IADL measure, a dimension was scored if the person regularly performed the activity in the past as a common part of their daily activity. 

C-reactive protein (CRP) was measured, and CRP > 5 mg/L defined inflammation.

### 2.6. Statistical Analysis

Qualitative variables were expressed as frequencies and percentages. Continuous variables were expressed as median with interquartile range (IQR).

Analyses were conducted separately in each group of patients; i.e., hospitalized in MCU, GCU, or GRU, or NH residents. We chose to differentiate patients in MCU and GCU, as healthcare providers in GCUs are trained in good clinical practices for geriatric care. Moreover, patients admitted into a GCU have more comorbidities than patients admitted into an MCU. 

Descriptive analyses were performed to assess sociodemographic and clinical characteristics. Energy and protein gaps were calculated as requirements minus intakes [25], with positive values indicating that intakes were lower than requirements. Protein intake was expressed in grams and as percentage of daily energy intake, which is the ratio of protein energy to total energy (P/E ratio). Intakes were compared to requirements using the Wilcoxon signed rank test. Intakes-to-requirements were compared according to malnutrition status using the non-parametric Mann–Whitney test. Spearman’s correlation coefficient was used to correlate intake-to-requirement gaps with age, BMI, daily oral medication, CIRS-G scores, ADL scores, and IADL scores. Energy and protein gaps were compared according to gender, life situation, inflammation, and sarcopenia, using the non-parametric Mann–Whitney test. 

Two-sided *p*-values < 0.05 were considered statistically significant. Statistical analyses were performed using SAS statistics software package v 9.4 (SAS Institute Inc., Cary, NC, USA).

## 3. Results

### 3.1. Partcipant Characteristics

The study included a total of 360 participants: 104 in the MCU group (women: 56.3%), 119 in the GCU group (women: 76.5%), 83 in the GRU group (women: 69.9%), and 54 in the NH group (women: 74.1%) (Table 1). The median participant age was 86.0 years (IQR 82.0–89.5) in the MCU group, 86.0 years (IQR 83.0–89.0) in the GCU group, 87.0 years (IQR 82.0–89.0) in the GRU group, and 88.0 years (IQR 85.0–93.0) in the NH group. Analysis of nutritional status found that more than 30% of all inpatients and NH residents were malnourished. Prevalence of malnutrition was 35.2% (95% CI: 25.4–45.0) in the MCU, 40.0% (95% CI: 30.8–49.2) in the GCU, 30.6% (95% CI: 19.9–41.2) in the GRU, and 31.3% (95% CI: 18.1–44.4) in the NH. Prevalence of severe malnutrition was 18.7% (95% CI: 10.7–26.7) in the MCU, 23.6% (95% CI: 15.7–31.6) in the GCU, 15.3% (95% CI: 7.0–23.6) in the GRU, and 4.2% (95% CI: 0.0–9.8) in the NH (Table 1).

### 3.2. Energy and Protein Intakes-to-Requirements

Complete daily energy and protein intake records were available for 36.5% of MCU patients (n = 38), 89.1% of GCU patients (n = 106), 66.3% of GRU patients (n = 55), and 68.5% of NH residents (n = 37). Participants with measured intakes did not have a significantly different malnutrition status to the other participants (*p* = 0.636 in the MCU group, *p* = 0.760 in the GCU group, *p* = 0.718 in the GRU, group, and *p* = 1.000 in the NH group). In the MCU group, patients with measured intakes needed more help with feeding than patients without measured intakes (36.4% vs. 13.3%, *p* = 0.010). In the GRU group, patients with measured intakes needed less help with feeding (16.4% vs. 46.4%, *p* = 0.003) and had more inflammation (78.2% vs. 55.6%, *p* = 0.034) than patients without measured intakes. GCU inpatients and NH residents with measured intakes were not significantly different to participants without measured intakes. 

**Figure 1:**
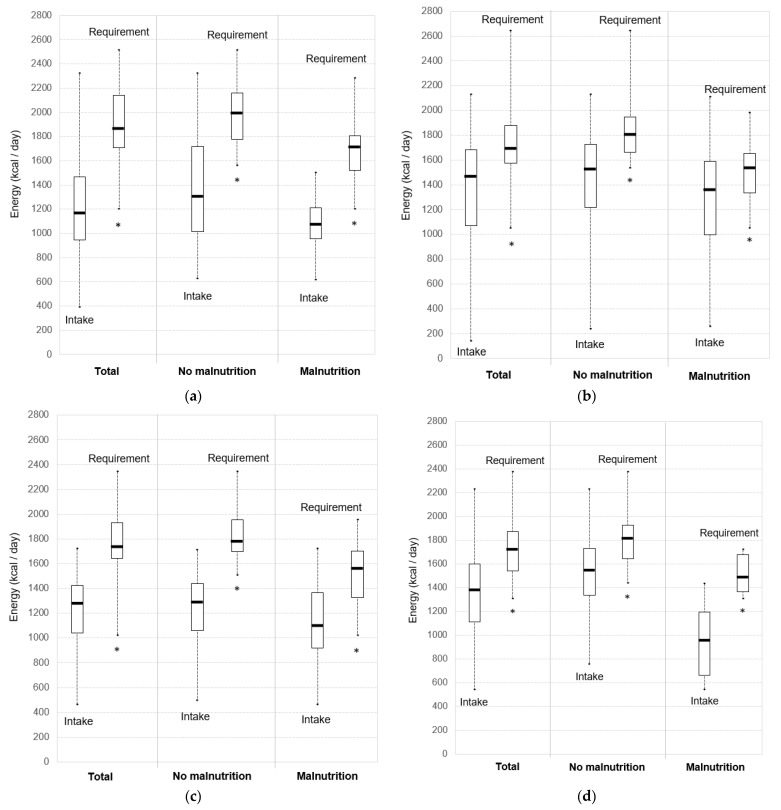
Daily energy intakes versus minimum requirements (30 kcal/kg/day): (**a**) Multidisciplinary Care Unit (MCU); (**b**) Geriatric Care Unit (GCU); (**c**) Geriatric Rehabilitation Unit (GRU); (**d**) Nursing Homes (NH). Boxplots indicate lower quartile, median, and upper quartile, and whiskers indicate minimum and maximum values. * Intakes significantly lower than requirements.

**Figure 2:**
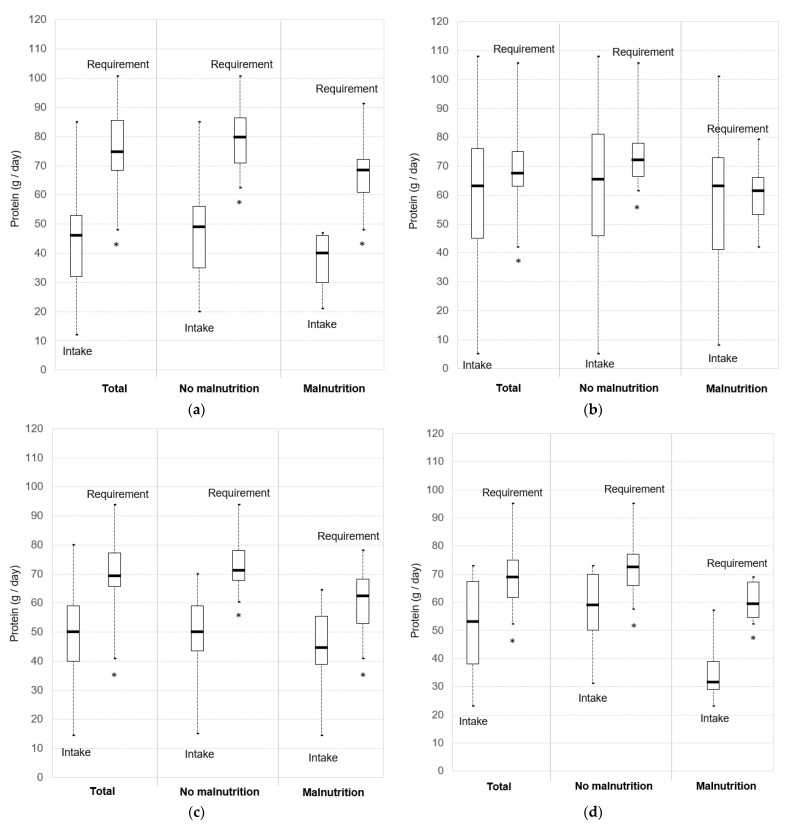
Daily protein intakes versus minimum requirements (1.2 g/kg/day): (**a**) Multidisciplinary Care Unit (MCU); (**b**) Geriatric Care Unit (GCU); (**c**) Geriatric Rehabilitation Unit (GRU); (**d**) Nursing Homes (NH). Boxplots indicate lower quartile, median, and upper quartile, and whiskers indicate minimum and maximum values. * Intakes significantly lower than requirements.

Daily energy and protein intakes of participants were compared to the minimum energy (Figure 1) and protein (Figure 2) requirements set out by French recommendations.

Energy intakes were lower than the minimum energy requirement of 30 kcal/kg/day in at least 75% of the total participant population; i.e., 89.5% of MCU patients, 75.5% of GCU patients, 92.7% of GRU patients, and 83.8% of NH residents. All participants in all settings had intakes under the maximum energy allowance of 40 kcal/kg/day, except in the GCU group (98.1% of patients). Energy intakes were significantly lower than the minimum requirements of 30 kcal/kg/day in all groups (*p* < 0.001) (Figure 1). Median gaps between daily energy intakes and requirements (based on a minimum energy requirement of 30 kcal/kg/day) were 680.0 kcal (IQR: 280.0–948.4) in the MCU group, 308.4 kcal (IQR: 6.4–655.0) in the GCU group, 536.5 kcal (IQR: 261.5–765.0) in the GRU group and 385.0 kcal (IQR: 125.7–755.1) in the NH group. 

Protein intakes were lower than the minimum protein requirement of 1.2 g/kg/day in a vast majority of participants; i.e., 100% of MCU patients, 64.2% of GCU patients, 90.9% of GRU patients, and 83.8% of NH residents. All participants in all settings had intakes under the maximum protein requirement of 1.5 g/kg/day, except in the GCU group (84.0% of patients). Protein intakes were significantly lower than the minimum requirement of 1.2 g/kg/day in all groups (*p* < 0.001) (Figure 2). Median gaps between daily protein intakes and requirements (based on a minimum protein requirement of 1.2 g/kg/day) were 32.9 g (IQR: 18.4–42.4) in the MCU group, 6.0 g (IQR: −7.8–22.1) in the GCU group, 20.4 g (IQR: 9.9–31.8) in the GRU group, and 17.2 g (IQR: 5.7–31.4) in the NH group. 

Median P/E ratios were 14.2% (IQR: 12.7–15.4) in the MCU group, 17.9% (IQR: 16.2–19.4) in the GCU group, 15.6% (IQR: 14.3–17.5) in the GRU group, and 15.6% (IQR: 13.1–17.0) in the NH group.

### 3.3. Intake-to-Requirement Gaps and Malnutrition

Energy intakes were significantly lower than the minimum requirement of 30 kcal/kg/day both for non-malnourished patients (*p* < 0.001 in MCU, GCU and GRU groups, and *p* = 0.006 in the NH group) and malnourished patients (*p* < 0.001 in the MCU group, *p* = 0.015 in the GCU group, *p* = 0.003 in the GRU group, and *p* = 0.002 in the NH group) (Figure 1). Energy gaps between intakes and requirements (based on a minimum energy requirement of 30 kcal/kg/day) were not significantly different between malnourished and non-malnourished participants, except in the GCU group (Figure 1 and Table 2). Non-malnourished patients in GCU had higher energy intake-to-requirement gaps than malnourished patients (*p* = 0.032) (Table 2). NH residents tended to have higher energy intake-to-requirement gaps if they were malnourished (*p* = 0.091). 

Protein intakes were significantly lower than the minimum requirement of 1.2 g/kg/day for non-malnourished patients (*p* < 0.001 in all groups) (Figure 1). Protein intakes of malnourished patients were significantly lower than requirement, except in the GCU group (*p* = 0.663) (*p* < 0.001 in the MCU group, *p* = 0.007 in the GRU group, and *p* = 0.004 in the NH group) (Figure 1). Protein intake-to-requirement gaps (based on a minimum protein requirement of 1.2 g/kg/day) and P/E ratios were not significantly different between malnourished and well-nourished participants (Table 2).

### 3.4. Factors Associated with Energy and Protein Intake-to-Requirement Gaps

In the MCU group, energy intake-to-requirement gaps (based on a minimum energy requirement of 30 kcal/kg/day) were significantly higher in men and were associated with more comorbidities and a lower ADL score (Table 3). MCU patients with probable sarcopenia had significantly higher energy and protein intake-to-requirement gaps. Protein gaps tended to increase with BMI (*p* = 0.078), more comorbidities (*p* = 0.060 for CIRS-G total score), and inflammation (*p* = 0.071). 

In the GCU group, energy and protein gaps were higher in men and in patients not living alone, but the difference was only significant (*p* < 0.05) for energy gaps (Table 4). GCU patients who needed help with feeding had significantly higher energy gaps. Patients with inflammation had significantly higher energy and protein gaps. 

In the GRU group, higher energy and protein intake-to-requirement gaps were associated with higher CIRS-G total comorbidity score and severity index, but the difference was only significant (*p* < 0.05) for protein gap (Table 5). Energy and protein gaps increased with CIRS-G comorbidity index, without statistical significance. GRU patients with inflammation had significantly higher energy and protein intake-to-requirement gaps. 

In the NH group, only lower daily functional ability in ADL was associated with higher energy and protein intake-to-requirement gaps, but the difference was only significant (*p* < 0.05) for energy gap (Table 6). 

## 4. Discussion

This study found that older-adult energy and protein intakes systematically fell short of energy and protein requirements in both hospital and nursing-home settings. Malnutrition status was not associated with the extent of the energy/protein gaps, except in the GRU setting where energy gaps were higher in patients without malnutrition. The factors identified as associated with higher energy gaps were gender, lower daily functional ability, more comorbidities, inflammation, not living alone, needing help with feeding, and probable sarcopenia. The factors identified as associated with higher protein gaps were more comorbidities, inflammation, and probable sarcopenia.

Computed results based on the five studies that had previously estimated dietary provision of nutritional requirements found that 49% to 82.7% of patients had an energy gap and 37% to 74% of patients had a protein gap [13,14,15,17,19]. Our study revealed that intake-to-requirement gaps were higher in the older-adult population. Only one previous study identified factors associated with intake-to-requirement gaps in older adults [17]. It found that age, middle-upper arm circumference, total arm area, albumin, C-reactive protein (CRP), CRP/albumin ratio, and impaired self-feeding at admission were associated with energy intake-to-requirement gaps in acute wards only [17]. It also showed that age, middle-upper arm circumference, total arm area, albumin, C-reactive protein (CRP), lymphocytes, CRP/albumin ratio, and impaired self-feeding at admission were associated with protein intake-to-requirement gaps [17]. Here we identified two comparative factors; i.e., inflammation, and probable sarcopenia, which is close to middle-upper arm circumference as another way to evaluate muscle. 

How can we increase intakes in hospitals and in NH? Protein and energy supplementation could improve intakes [32,33]. Several studies have proposed to address malnutrition through various strategies, such as implementing volunteer mealtime assistants [34,35,36,37], improved meal presentation [38,39], room service [40], electronic beside meal ordering [41,42], fortified-food meals [32], or managing the barriers to food intake interruptions at meals [43]. The European Society for Clinical Nutrition and Metabolism (ESPEN) has issued recommendations advocating better integration of aging-related needs in hospitalization, including systematic screening and earlier management of malnutrition, performing training exercises at the beginning of the hospital stay, and better preparing patient discharge to avoid premature readmissions [2]. 

A new paradigm is needed concerning nutritional care in hospitals and NH. The current nutritional care paradigm is based on one portion of standardized enriched and high-protein meal, irrespective of the patient’s characteristics. First, all older adults should benefit from enriched meals straight from admission to hospital, as they have a high prevalence of malnutrition and their bodies have to contend with acute disease. Second, meal portions should also be adapted. For example, a malnourished older adult will receive greater benefit from a half portion of a high-protein meal than a full portion of an enriched meal, due to their low BMI and low appetite. A patient-centered approach should be developed based on calculating the patient’s specific health status-related requirements (age, malnutrition, and sarcopenia status, obesity, appetite, chronic pathologies, pressure ulcer). Moreover, energy and protein intakes should be monitored precisely and daily, using a computerized device that can alert staff to situations where intakes fall short of requirements.

This study, performed in a multidepartment setting that spanned the continuum of older adult hospitalization, acute care, and rehabilitation wards, as well as a non-hospital living situation, had some limitations. Nutritional intakes were not measured in the whole sample, notably in the MCU. However, participants with measured intakes were not significantly different from other participants in terms of malnutrition status. Second, intakes were measured at the beginning of the hospital stay and during the next 3 days, which corresponded to the worst health state and therefore could have led to overestimate the gaps between intakes and requirements. Intakes should be measured throughout the hospital stay, which could be done using electronic devices. 

## 5. Conclusions

Energy and protein intakes fell dramatically short of energy and protein requirements in older adult hospitalization settings and nursing homes irrespective of malnutrition status. Long-term monitoring of inpatient and resident intakes should be performed to make healthcare professionals more systematically aware of malnutrition issues. Moreover, a new paradigm based on a patient-centered approach should be developed in order to adapt meals served meals to inpatients’ and residents’ physical characteristics and/or food routines so as to prevent or at least not worsen malnutrition.

## Figures and Tables

**Table 1:** Sociodemographic and clinical characteristics of participants.

Characteristics	Multidisciplinary Care Unit (MCU)	Geriatric Care Unit (GCU)	Geriatric Rehabilitation Unit (GRU)	Nursing Homes (NH)
Participants, n	104	119	83	54
Age (years), median (IQR)	86.0 (82.0–89.5)	86.0 (83.0–89.0)	87.0 (82.0–89.0)	88.0 (85.0–93.0)
Women, n (%)	59 (56.7)	91 (76.5)	58 (69.9)	40 (74.1)
BMI (kg/m^2^), median (IQR)	25.3 (22.2–29.7)	26.9 (22.5–31.3)	28.4 (24.4–32.2)	29.0 (24.5–32.5)
Living situation, n (%)				NA
At home	93 (89.4)	107 (89.9)	79 (95.2)	
Residential home	8 (7.7)	8 (6.7)	0 (0)	-
Other	3 (2.9)	4 (3.4)	4 (4.8)	-
Life situation, n (%)				NA
Living alone	60 (65.2)	77 (69.4)	56 (67.5)	-
With partner	18 (19.6)	22 (19.8)	22 (26.5)	-
With a family member	14 (15.2)	12 (10.8)	5 (6.0)	-
Daily medications, median (IQR)	8.0 (5.0–9.0)	7.0 (5.0–9.0)	8.0 (5.0–10.0)	9.0 (7.0–11.0)
CIRS-G, median (IQR)				
Total score	12.0 (9.0–15.0)	16.0 (12.0–20.0)	17.0 (12.0–22.0)	17.0 (14.0–21.0)
Severity index	0.9 (0.7–1.2)	1.1 (0.8–1.4)	1.2 (0.8–1.5)	1.2 (0.8–1.5)
Comorbidity index	4.0 (3.0–6.0)	5.0 (3.0–6.0)	5.0 (3.0–7.0)	6.0 (4.0–7.0)
ADL, median (IQR)	5.5 (3.5–6.0)	5.0 (4.0–6.0)	5.5 (4.5–6.0)	3.0 (1.5–4.8)
Needing help with feeding *, n (%)	20 (21.5)	24 (20.2)	22 (26.5)	20 (38.5)
IADL, median (IQR)	4.3 (2.0–7.0)	4.0 (3.0–6.0)	5.0 (3.0–6.9)	NA
Inflammation (CRP > 5), n (%)	69 (67.0)	83 (69.7)	58 (70.7)	28 (53.8)
Sarcopenia, n (%)				
Probable	51 (51.0)	74 (66.7)	52 (69.3)	35 (72.9)
Confirmed	18 (20.2)	35 (32.1)	17 (25.0)	13 (28.9)
Malnutrition, n (%)	32 (35.2)	44 (40.0)	22 (30.6)	15 (31.3)
Stage 1—Moderate	15 (16.5)	18 (16.4)	11 (15.3)	13 (27.1)
Stage 2—Severe	17 (18.7)	26 (23.6)	11 (15.3)	2 (4.2)

NA: Not applicable; CIRS-G: Cumulative Illness Rating Scale-Geriatric; ADL: Activities of Daily Living; IADL: Instrumental Activities of Daily Living; IQR: Inter Quartile range. * ADL feeding dimension.

**Table 2:** Gaps between energy and protein intakes and minimum requirements and P/E ratios stratified by malnutrition status.

Characteristics	Multidisciplinary Care Unit (MCU)	Geriatric Care Unit (GCU)	Geriatric Rehabilitation Unit (GRU)	Nursing Homes (NH)
No.	34	98	48	33
Energy gap (kcal), median (IQR)				
Malnutrition	592.5 (220.0–770.0)	160.0 (−174.0–543.0)	504.3 (122.0–736.0)	640.8 (301.0–765.5)
No malnutrition	650.0 (280.0–932.0)	317.1 (106.4–637.9)	598.1 (370.1–769.5)	268.6 (−5.6–617.0)
*p*-value	0.860	**0.032**	0.231	0.091
Protein gap (g), median (IQR)				
Malnutrition	32.2 (16.4–41.8)	1.4 (−15.4–20.4)	18.5 (0.7–30.4)	24.7 (17.6–36.1)
No malnutrition	33.0 (20.9–42.4)	7.1 (−2.6–22.1)	21.0 (13.9–33.0)	12.5 (4.2–26.0)
*p*-value	0.833	0.116	0.199	0.106
P/E ratio (%), median (IQR)				
Malnutrition	14.2 (13.2–15.0)	17.9 (16.2–19.1)	16.9 (14.9–17.5)	14.5 (12.9–18.2)
No malnutrition	14.2 (12.7–15.7)	18.1 (16.6–19.5)	15.4 (14.3–17.5)	15.7 (13.9–16.4)
*p*-value	0.888	0.663	0.431	0.574

Significant *p*-values are reported in bold text (*p* < 0.05). Energy gap: requirement of 30 kcal/kg/day—intake; protein gap: requirement of 1.2 g/kg/day—intake; P/E ratio: protein energy/total energy. A positive gap value indicates intake lower than requirements.

**Table 3:** Factors associated with energy and protein intake-to-requirement gaps in the Multidisciplinary Care Unit group.

Multidisciplinary Care Unit	Energy Gap	*p*-Value	Protein Gap	*p*-Value
Age (years), *r*	0.05	0.748	0.02	0.908
Gender, median (IQR)		**0.034**		0.122
Men	882.2 (468.3–1162.4)		35.8 (28.1–46.8)	
Women	584.8 (220.0–766.0)		27.6 (17.6–39.7)	
BMI (kg/m^2^), *r*	0.20	0.235	0.30	0.078
Life situation, median (IQR)		0.517		0.427
Living alone	616.9 (280.0–816.0)		28.4 (18.4–40.0)	
With partner or a family member	710.0 (190.5–1277.1)		34.0 (15.6–55.0)	
Daily medications, *r*	0.14	0.398	0.15	0.384
CIRS-G, *r*				
Total score	0.34	**0.047**	0.32	0.060
Severity index	0.27	0.102	0.27	0.096
Comorbidity index	0.21	0.202	0.29	0.079
ADL, *r*	−0.35	**0.048**	−0.29	0.102
Needing help with feeding *, median (IQR)		0.956		0.566
No	650.0 (220.0–1052.0)		33.0 (17.6–46.5)	
Yes	679.3 (325.3–944.5)		31.1 (23.6–37.3)	
IADL, *r*	−0.28	0.148	−0.26	0.185
Inflammation (CRP > 5), median (IQR)		0.347		0.071
No	596.9 (280.0–770.0)		27.3 (16.4–33.0)	
Yes	738.0 (278.0–1015.6)		37.7 (23.8–45.1)	
Probable sarcopenia, median (IQR)		**0.015**		**0.017**
No	504.3 (220.0–751.0)		26.3 (16.4–35.0)	
Yes	865.5 (592.5–1052.0)		39.2 (30.0–46.5)	
Confirmed sarcopenia, median (IQR)		0.497		0.404
No	617.5 (237.0–915.0)		30.0 (17.6–40.0)	
Yes	670.8 (271.8–1319.8)		37.0 (18.2–55.6)	

Data are presented as Spearman’s correlation coefficient (*r*) or median with interquartile range (IQR). Significant *p*-values are reported in bold text (*p* < 0.05). Energy gap: requirement of 30 kcal/kg/day—intake; protein gap: requirement of 1.2 g/kg/day—intake. A positive gap value indicate intake lower than requirements. CIRS-G: Cumulative Illness Rating Scale-Geriatric; ADL: Activities of Daily Living; IADL: Instrumental Activities of Daily Living. * ADL feeding dimension.

**Table 4:** Factors associated with energy and protein intake-to-requirement gaps in the Geriatric Care Unit group.

Geriatric Care Unit	Energy Gap	*p*-Value	Protein Gap	*p*-Value
Age (years), *r*	0.13	0.199	0.07	0.456
Gender, median (IQR)		**0.031**		0.053
Men	447.0 (234.0–1011.7)		15.9 (2.1–31.4)	
Women	233.0 (−21.0–564.0)		3.6 (−9.6–21.0)	
BMI (kg/m^2^), *r*	0.17	0.077	0.12	0.236
Life situation, median (IQR)		**0.031**		0.064
Living alone	238.5 (−19.0–542.0)		4.5 (−9.6–19.7)	
With partner or a family member	421.7 (233.0–1011.7)		9.0 (1.2–36.8)	
Daily medications, *r*	0.14	0.148	0.08	0.396
CIRS-G, *r*				
Total score	0.17	0.081	0.11	0.282
Severity index	0.17	0.088	0.10	0.291
Comorbidity index	0.16	0.112	0.11	0.268
ADL, *r*	−0.16	0.113	−0.11	0.247
Needing help with feeding *, median (IQR)		**0.033**		0.161
No	271.0 (−19.0–496.0)		4.9 (−7.8–21.1)	
Yes	655.0 (65.7–1011.7)		19.7 (−3.0–36.8)	
IADL, *r*	−0.10	0.324	−0.11	0.267
Inflammation (CRP > 5), median (IQR)		**0.001**		**0.034**
No	20.5 (−162.0–435.0)		−2.4 (−12.8–18.2)	
Yes	408.6 (196.5–732.2)		8.7 (−0.9–25.4)	
Probable sarcopenia, median (IQR)		0.683		0.791
No	314.5 (5.5–511.1)		7.0 (−7.9–16.7)	
Yes	265.5 (−12.0–662.0)		4.1 (−8.1–25.4)	
Confirmed sarcopenia, median (IQR)		0.359		0.648
No	304.0 (49.0–560.6)		5.5 (−5.0–21.0)	
Yes	234.0 (−165.0–655.0)		3.1 (−12.8–21.4)	

Data are presented as Spearman’s correlation coefficient (*r*) or median with interquartile range (IQR). Significant *p*-values are reported in bold text (*p* < 0.05). Energy gap: requirement of 30 kcal/kg/day—intake; protein gap: requirement of 1.2 g/kg/day—intake. A positive gap value indicates intake lower than requirements. CIRS-G: Cumulative Illness Rating Scale-Geriatric; ADL: Activities of Daily Living; IADL: Instrumental Activities of Daily Living. * ADL feeding dimension.

**Table 5:** Factors associated with energy and protein intake-to-requirement gaps in the Geriatric Rehabilitation Unit group.

Geriatric Rehabilitation Unit	Energy Gap	*p*-Value	Protein Gap	*p*-Value
Age (years), *r*	−0.02	0.908	0.08	0.584
Gender, median (IQR)		0.228		0.382
Men	664.0 (487.9–765.0)		21.9 (13.8–38.4)	
Women	526.9 (213.0–731.0)		20.3 (8.7–30.4)	
BMI (kg/m^2^), *r*	0.17	0.221	0.14	0.325
Life situation, median (IQR)		0.821		0.898
Living alone	575.0 (329.0–769.5)		21.0 (8.7–31.8)	
With partner or a family member	525.3 (234.0–736.0)		20.4 (13.9–21.6)	
Daily medications, *r*	0.12	0.392	0.04	0.751
CIRS-G, *r*				
Total score	0.26	0.058	0.28	**0.038**
Severity index	0.26	0.055	0.28	**0.038**
Comorbidity index	0.24	0.082	0.25	0.063
ADL, *r*	0.05	0.702	0.02	0.876
Needing help with feeding *, median (IQR)		0.549		0.316
No	532.5 (329.0–731.0)		20.0 (9.9–31.6)	
Yes	664.0 (170.0–910.0)		24.2 (17.3–34.3)	
IADL, *r*	0.01	0.942	−0.09	0.519
Inflammation (CRP > 5), median (IQR)		**0.027**		**0.027**
No	385.2 (58.0–546.1)		11.9 (−1.3–23.1)	
Yes	602.1 (340.9–798.5)		21.2 (13.9–33.7)	
Probable sarcopenia, median (IQR)		0.838		0.599
No	466.0 (340.9–694.5)		20.6 (17.3–31.6)	
Yes	575.0 (189.0–781.8)		19.4 (8.2–33.6)	
Confirmed sarcopenia, median (IQR)		0.173		0.123
No	598.1 (370.1–769.5)		21.0 (13.9–33.0)	
Yes	480.0 (122.0–736.0)		18.0 (0.7–30.4)	

Data are presented as Spearman’s correlation coefficient (*r*) or median with interquartile range (IQR). Significant *p*-values are reported in bold text (*p* < 0.05). Energy gap: requirement of 30 kcal/kg/day—intake; protein gap: requirement of 1.2 g/kg/day—intake. A positive gap value indicates intake lower than requirements. CIRS-G: Cumulative Illness Rating Scale-Geriatric; ADL: Activities of Daily Living; IADL: Instrumental Activities of Daily Living. * ADL feeding dimension.

**Table 6:** Factors associated with energy and protein intake-to-requirement gaps in the Nursing Homes group.

Nursing Homes	Energy Gap	*p*-Value	Protein Gap	*p*-Value
Age (years), *r*	0.07	0.673	0.01	0.966
Gender, median (IQR)		0.095		0.164
Men	767.0 (197.2–941.6)		24.3 (11.1–37.9)	
Women	357.3 (89.0–613.0)		16.0 (4.2–26.0)	
BMI (kg/m^2^), *r*	−0.08	0.629	−0.15	0.365
Daily medications, *r*	0.00	0.983	−0.03	0.883
CIRS-G, *r*				
Total score	0.20	0.244	0.16	0.339
Severity index	0.22	0.198	0.17	0.324
Comorbidity index	0.16	0.357	0.10	0.540
ADL, *r*	−0.35	**0.040**	−0.29	0.088
Needing help with feeding *, median (IQR)		0.186		0.499
No	273.5 (−3.9–564.0)		14.6 (1.2–29.1)	
Yes	563.7 (140.7–765.5)		17.6 (8.2–26.1)	
Inflammation (CRP > 5), median (IQR)		0.247		0.489
No	246.0 (89.0–474.5)		13.2 (5.7–25.0)	
Yes	562.9 (140.7–759.0)		16.8 (4.2–35.7)	
Probable sarcopenia, median (IQR)		0.411		0.482
No	275.1 (−5.6–474.5)		12.1 (5.1–26.0)	
Yes	330.5 (89.0–758.3)		17.4 (4.2–35.7)	
Confirmed sarcopenia, median (IQR)		0.276		0.258
No	272.3 (−5.6–617.0)		14.2 (5.1–26.0)	
Yes	562.0 (246.0–759.0)		26.1 (13.2–36.1)	

Data are presented as Spearman’s correlation coefficient (*r*) or median with interquartile range (IQR). Significant *p*-values are reported in bold text (*p* < 0.05). Energy gap: requirement of 30 kcal/kg/day—intake; protein gap: requirement of 1.2 g/kg/day—intake. A positive gap value indicates intake lower than requirements. CIRS-G: Cumulative Illness Rating Scale-Geriatric; ADL: Activities of Daily Living; IADL: Instrumental Activities of Daily Living. * ADL feeding dimension.

## Data Availability

The data that support the findings of this study are available from the corresponding author upon reasonable request.
